# To hug or not to hug? Public and private displays of affection and relationship satisfaction among people from Indonesia, Nepal, and Poland

**DOI:** 10.1371/journal.pone.0326115

**Published:** 2025-06-25

**Authors:** Dagna Kocur, Łukasz Jach, Magdalena Sitko-Dominik, Sandesh Dhakal, Nandita Sharma, Yudi Tri Harsono, Irena Przybylska, Edyta Nieduziak, Daniela Dzienniak-Pulina, Agnieszka Rychłowska-Niesporek, Dawid Zozgórnik

**Affiliations:** 1 Faculty of Social Sciences, Institute of Psychology, University of Silesia in Katowice, Katowice, Poland; 2 Central Department of Psychology, Tribhuvan University, Kirtipur, Kathmandu, Nepal; 3 Department of Psychology, State University of Malang, Kota Malang, Java Timur, Indonesia; 4 Faculty of Social Sciences, Institute of Pedagogy, University of Silesia in Katowice, Katowice, Poland; 5 Faculty of Social Sciences, Institute of Sociology, University of Silesia in Katowice, Katowice, Poland; James Cook University, SINGAPORE

## Abstract

Displays of romantic affection, both public and private, contribute to improved well-being, reduced stress, greater life satisfaction, and enhanced relationship satisfaction. However, cultural norms significantly influence the expression of affection, particularly in public settings. This study aimed to compare individuals from Indonesia, Nepal, and Poland regarding their public and private displays of affection, negative attitudes, and behaviors towards other individuals engaging in public displays of affection. Additionally, we examined relationship satisfaction and its associations with the expression of affection. We collected data from 170 Indonesian participants (62 men and 108 women), 120 Nepali participants (56 men and 64 women), and 171 Polish participants (72 men and 99 women) aged 18 to 49. The results revealed cultural differences in public displays of affection: Polish participants reported the highest level, while Indonesian participants reported the lowest. Differences also emerged for private displays of affection: Polish participants scored higher than Nepali and Indonesian participants, while no differences were observed between participants from Nepal and Indonesia. Negative attitudes and behaviors towards displaying affection publicly were most prevalent in Indonesia and least prevalent in Poland. Nepali participants reported the highest level of relationship satisfaction, followed by Polish participants, with Indonesian participants reporting the lowest. Across all three countries, positive correlations were observed between displays of affection and relationship satisfaction, underscoring the importance of affectionate behaviors in romantic relationships. Moreover, our findings highlight the influence of cultural norms on the expression of romantic affection and suggest that relationship satisfaction is closely tied to affectionate behaviors, albeit with variations shaped by cultural context.

## 1. Introduction

Displays of romantic affection, both public and private, are important for better well-being, lower stress, greater life satisfaction [1], and greater relationship satisfaction [2]. However, the opportunity to express romantic affection is regulated by ethnicity, culture, and religion [3–5]. In some countries (e.g., USA, Canada, European Union countries, Australia, and New Zealand), people can freely show romantic affection in public; on the other hand, in other countries (e.g., Pakistan, Iraq, Saudi Arabia, Jordan, and Egypt) public expression of romantic feelings is a social taboo. Moreover, legal regulations in some countries prohibit romantic displays of affection in public (e.g., the United Arab Emirates, India, and Saudi Arabia). On the other hand, even in countries with very conservative cultures, the rules in the public and in the private sphere may differ [6,7]. However, to date, no studies have explored how differences in the willingness to show affection publicly and privately in heterosexual relationships are related to relationship satisfaction. We aimed to address this gap by analyzing data from Indonesia, Nepal, and Poland. This study contributes to a better understanding of how cultural norms may influence the expression of romantic affection in different relational contexts and how such expressions relate to relationship satisfaction across diverse cultures. Understanding these differences is important, as the ability to express affection in ways that align with cultural expectations can influence the quality and stability of romantic relationships and individual health and well-being. This may be particularly relevant in regions where cultural norms discourage the open expression of romantic affection.

However, to date, no studies have explored how differences in the willingness to publicly and privately display affection in heterosexual relationships are associated with relationship satisfaction. We aimed to address this gap by analyzing data from Indonesia, Nepal, and Poland.

### 1.1. Display of affection

Affection is “a feeling of warmth and fondness toward someone” [8, p. 59]. In turn, affectionate communication is defined as “an individual’s intentional and overt enactment or expression of feelings of closeness, care, and fondness for another” [9], p. 145. Communicating feelings is a common behavior [10] and behaviors aimed at showing feelings are presented in relationships ranging from casual acquaintances to intimacy [11].

However, many studies have distinct differences in defining affection. For example, affection is sometimes operationalized as kissing frequency or affectionate touch [1], and in other cases, it means words of appreciation, physical touch, doing favors for the partner, giving gifts, and spending quality time with the partner [12]. Gulledge et al. [13] list seven behaviors related to showing affection: holding hands, kissing on the lips, placing kisses on the partner’s face, hugging, massaging, cuddling, and caressing. According to Floyd et al. [10], these behaviors include: verbal declarations of love and care, hugging, kissing, complimenting and encouraging, listening and responding, providing help, undertaking joint activities, cooking, touching, engaging in conversations on important topics, holding hands, flirting, and engaging in sexual activity. In turn, Vaquera and Kao [14] differentiate affection behaviors depending on the context in which they are communicated: public, private, and intimate.

The two-dimensional model of expressing feelings distinguished verbal and nonverbal behaviors [15]. On the other hand, the tripartite model of communicating feelings [9] also indicated direct and indirect behaviors. Therefore, the following types of behaviors aimed at expressing feelings were distinguished: direct and verbal (e.g., telling someone that we like them), direct and nonverbal (e.g., smiling at someone), and indirect and nonverbal (e.g., providing support to someone in completing tasks) [16].

The Affection Exchange Theory (*AET*), developed by Floyd [17], emphasizes the role of affectionate communication as an adaptive behavior that supports survival and reproductive success. Floyd argues that affection is key to forming and maintaining social bonds, which increase the chances of survival for individuals and their offspring. Affection promotes pair bonding, providing access to resources and enhancing reproductive attractiveness. *AET* is grounded in Darwinian evolutionary principles, positing that the expression of affection is a natural selection mechanism that influences social, emotional, and biological development [18]. Floyd’s research highlights individual genetic and neurological differences, as well as gene-environment interactions in the expression of affection [19]. Additionally, affection deprivation is strongly associated with negative health consequences, such as depression, loneliness, stress, and sleep problems [20]. Studies also demonstrate that affection deprivation has a more harmful impact on health than excessive affection. Furthermore, expressing affection in romantic relationships is closely linked to relationship satisfaction [21].

Gender is important in shaping how individuals express affection in romantic relationships. Women tend to show affection more frequently than men [7,22], while men are more likely to report a lack of affectionate touch and greater affection deprivation [23]. Although men and women differ in their preferred forms of physical affection—men often prefer kissing, while women favor hugging—these differences are generally modest [24].

### 1.2. Display of affection and relationship satisfaction

According to the Affection Exchange Theory [17], humans express affection to increase their survival and reproduction in social contexts. Moreover, receiving affection is pleasurable and improves well-being [25]. Positive links of romantic affection towards a partner with relationship satisfaction and relationship quality were reported in many studies [2,24]. Moreover, the Actor Partner Interdependence Model analyses revealed that the actor’s expression of affection significantly affects the satisfaction of both the actor and the partner [26] and the phenomenon of increasing relationship satisfaction by expressing and receiving affection was confirmed experimentally [2,27]. On the other hand, greater satisfaction with the relationship may motivate people to show their affection [28]. When comparing the significance of public and private displays of affection, private displays, both one’s own and those expressed by a partner, are more important for relationship satisfaction and sexual satisfaction than public displays of affection [7].

Numerous mechanisms regulate the associations between displays of affection and relationship satisfaction. Hand-holding mitigates physiological stress during couple conflict discussions [29]; therefore displays of affection may enhance coping with stress [2]. Affection is also positively associated with the willingness to compromise [30], better communication during conflict [29,31], and better conflict resolution [24]. On the other hand, a higher frequency of affection is associated with feelings of greater intimacy [32,33] and positively affects commitment to the relationship [34]. Moreover, people who receive affection report fewer ruminations and feelings of harm [35].

### 1.3. Display of affection across cultures

Feelings are internal experiences; however, their external expression is subject to socially learned display rules that regulate the communication of emotions within a cultural context [36] and determine appropriate ways of communicating emotions [37]. Culturally diverse approaches to public displays of affection are described within the framework of Hofstede’s Cultural Dimensions Theory, which compares cultures across such dimensions as individualism, power distance, uncertainty avoidance, masculinity versus femininity, long-term orientation, and indulgence [38]. In our study, we focused exclusively on the cultural dimension of individualism, as it has been consistently linked to differences in emotional expression. People from individualistic cultures express emotions more frequently and feel more comfortable doing so than people from collectivistic cultures [36,39]. Indonesia, Nepal, and Poland differ in the mentioned cultural dimensions, particularly in terms of individualism. The levels of individualism are as follows: Indonesia: 14 [40]; Nepal: 30 [41]; Poland: 60 [42], on a scale from 1 to 100 with higher scores indicating greater cultural individualism. Moreover, Indonesia, Nepal, and Poland differ in religiosity and the dominant religions practiced, which may influence attitudes towards public displays of affection. These cultural and religious contexts shape norms around expressing emotions publicly and may affect the readiness to engage in such behaviors.

For Indonesian people, intimacy is a form of expression of affection that should be done in a private place and is always assessed from a religious point of view. The majority of the Indonesian population is Islamic, with religious rules affecting the cultural perspective and behaviors regarding public display of affection. In conservative regions such as Aceh, where Sharia law applies, public displays of affection like hugging or holding hands between unmarried couples can result in public punishment, including flogging. Even in more urban areas, such behaviors are often met with disapproval, reflecting the prevailing societal focus on modesty and propriety. In Islamic contexts, public displays of romantic affection are generally discouraged due to religious norms emphasizing modesty and appropriate conduct. In some regions, such displays may even be legally sanctioned [43]. Studies have shown that religiosity in Indonesian youth tends to postpone their involvement in romantic relationships [44]. Moreover, for married couples, kissing is allowed and justified; however, not everything that is allowed and justified can be shown or exhibited in the presence of other people [6]. Kissing in public is considered inappropriate, especially among unmarried couples [45]. Marital status is also a benchmark of tolerance for people who display their affection in public, even though married couples still must pay attention to the applicable norms. In addition, both uploading photos and videos that show intimacy such as kissing a spouse is taboo [6]. Among Indonesian adolescent Facebook users, public display of affection decreases reputation because people do not like to open themselves up to intimate matters; therefore, public display of affection provokes uncomfortable reactions, irritation, and disgust, and decreases liking for a person behaving in this way [46].

For a long time, Nepali society has been conservative regarding public displays of affection. Hinduism and Buddhism, the two major religions embraced by Nepali people, do not encourage public display of feelings in a romantic relationship. Although such display is not usually considered illegal or sinful, it is considered shameful. Traditionally, Hinduism has been accepting of romantic love and affection, as seen in the worship of Kamadeva, the god of love, and depictions of love in ancient art, such as in the Khajuraho temples [47]. However, contemporary right-wing Hindu groups, resisting Western influences, have opposed public displays of affection, considering them immoral and contrary to traditional values [48]. Despite historical acceptance, today public displays of affection are largely taboo in Hinduism due to the influence of conservative ideologies [49,50]. People in public settings are expected to be modest and restrained in showing romantic affection. A study conducted among married couples from the Newar community revealed disapproval towards public display of affection regardless of gender and the type of marriage; moreover, the participants were concerned that their children would adopt such behavior before reaching maturity [51]. On the other hand, young people are becoming favorable towards dating, but that is limited to private contexts [52] and even emerging adults who accept dating and romantic interactions consider public displays of affection inappropriate [53].

Among European countries, Poland is often perceived as rather conservative, and also as a place where the Catholic religion plays a significant role in shaping social norms [54].In Poland, where Catholicism is the dominant religion, the Catholic Church plays a role in shaping societal attitudes towards romantic relationships and displays of affection. Traditionally, the Catholic Church promotes premarital chastity and abstinence, considering public displays of love, such as kissing or hugging, inappropriate, especially in public spaces. According to the teachings of the Catholic Church, sexuality and intimacy should be expressed within marriage, respecting the sanctity of the relationship [55]. While modern Polish society becomes increasingly diverse in its approach to love and intimacy, the Catholic Church maintains a conservative stance on public displays of affection, accepting romantic feelings within marriage but rejecting them in other contexts [56]. However, when compared to Indonesia and Nepal, Poland might be seen as a more liberal country. Moreover, people in Nepal and Indonesia, where religious and cultural norms are even stricter, often have more conservative views on personal freedom, gender roles, and sexual expression. Research on public display of affection in Poland revealed a correlation between the approval of a traditionalist worldview and a conservative dating style, which emphasizes restraint in public displays of affection, commitment to long-term relationships, control over sexual aspects, and adherence to traditional gender roles [57]. Moreover, acceptance of a postmodernist worldview was positively linked to public displays of affection and a preference for recreational dating styles [57]. Another study showed that although Polish people in heterosexual relationships are generally willing to express affection in public, they still show a higher preference to do so in private settings [7].

### 1.4. The current study

In our study, we aimed to compare tendencies toward romantic displays of affection in both private and public contexts among people engaged in heterosexual romantic relationships in Indonesia, Nepal, and Poland. These three countries represent cultures that differ in many respects. One key difference is their level of individualism: Indonesia scores 14, Nepal 30, and Poland 60 on Hofstede’s individualism index [40–42]. Thus, Nepal demonstrates twice the level of individualism compared to Indonesia, while Poland exhibits twice the level of individualism found in Nepal. Second, these countries differ in their dominant religions. In Indonesia, the predominant religion is Islam (87%), in Nepal it is Hinduism (81%), and in Poland it is the Catholic version of Christianity (94%) [58]. Each of these religions differs substantially in its stance on the acceptability of romantic displays of affection, both in public and private contexts. Third, traditional norms regarding romantic displays of affection also vary across these countries. These norms may stem more from local customs and cultural traditions than from religious affiliation or the level of individualism itself. For example, levels of individualism do not fully explain cross-cultural differences in emotional display rules [39]. Because displays of affection have been shown to influence relationship satisfaction [1,2,26,27], we also aimed to compare the associations between relationship satisfaction and affectionate behaviors in private and public contexts, to check whether one type of display may be more strongly related to satisfaction than the other.

Based on the assumptions of the Affection Exchange Theory [17], we predicted, first and foremost, cultural differences resulting from variations in individualism, religion, culture, and gene-environment interactions in the context of romantic expressions of affection [19]. Additionally, based on Hofstede’s Cultural Dimensions Theory [38,59], we hypothesized that particularly public displays of affection would depend on the level of individualism in a given country.

Our study was exploratory; however, in the context of previous research [1,2,26,27], we predicted positive associations between displays of affection and relationship satisfaction in all the countries compared (H1). Additionally, in the context of cultural dimensions theory [38,59] and cross-country differences in individualism among people from the compared countries [40–42], we predicted the highest preference for displays of affection among Polish participants and the lowest preference among Indonesian participants (H2).

## 2. Materials and methods

### 2.1. Participants and procedure

We collected samples of 170 Indonesian participants (62 men and 108 women), 120 Nepali participants (56 men and 64 women), and 171 Polish participants (72 men and 99 women) aged 18 to 49 (*M* = 23.70, *SD* = 5.13). Data collection was conducted from July 1 to August 31, 2024. Written consent was obtained from all individual participants. All participants were in a heterosexual romantic relationship. We conducted the study utilizing students as interviewers who invited the participants to join the research. Participation was voluntary, with no remuneration provided. We informed the participants about the nature of the study and assured them of their anonymity.

The study was approved by The University of Silesia Institutional Ethics Committee (approval number: KEUS508/05.2024). We report all procedures, including manipulations, measures, and exclusions, ensuring transparency and adherence to ethical research standards. Data and materials associated with the present study are openly available on the Open Science Framework: https://osf.io/rqdg2/?view_only=e9f891ac2ad447a79645a51ec2ad1c06. This study was not preregistered.

### 2.2. Measures

We measured displays of affection using the Public and Private Romantic Display of Affection Scale *PRDAS* by Kocur et al. [7] that addresses various ways of expressing feelings, for example holding hands, hugging, kissing, and verbal declarations of love. The *PPRDAS* consists of four scales measuring such aspects of displays of affection as private display of affection [five items; e.g., “I like holding my partner’s hand while at home (e.g., watching a film).”, McDonald’s ω in current study = .83], public display of affection [five items; e.g., “I like walking in public places holding my partner’s hand.”; ω = .75], negative opinions about people displaying affection in public [four items; e.g., “Couples holding hands in public places annoy me.”, ω = .68], and negative behaviors towards people displaying affection in public [five items; e.g., “I sometimes admonish couples kissing passionately in public places.”, ω = .80]. The participants responded on a Likert scale from 1 (*Completely does not match my behavior*) to 5 (*Completely matches my behavior*). We averaged scores for the corresponding items to create two indexes of willingness to display affection (e.g., in private context and public context) and two indexes of attitudes towards displaying affection in public (e.g., negative opinions and negative behaviors). We did not calculate any global scores aggregating the results of several scales.

To measure relationship satisfaction we used a single-item method and we asked the participants to evaluate the quality of their romantic relationship on a scale from 1 (very unsuccessful) to 5 (very successful).

### 2.3. Analyses design

To make sure that comparing the samples we collected in Indonesia, Nepal, and Poland makes sense at all, we first wanted to compare the demographic characteristics of these samples. Therefore, we planned to conduct a chi-square test to compare gender distributions and an ANOVA analysis to compare age both between the overall samples collected in the three countries and between men and women studied within the same country. To compare the levels of relationship satisfaction of men and women from Indonesia, Nepal, and Poland, we planned to conduct ANOVA analysis. On the other hand, to examine participants’ willingness to display affection we planned a mixed model ANOVA with sex and country as between-subject factors and context of displaying affection as a within-subject factor. Similarly, we planned to conduct a mixed-model ANOVA to compare participants’ attitudes towards displaying affection in public across attitude type (within-subject factor) and participants’ sex and country (between-subject factors). We applied the Bonferroni-Holm correction for multiple post-hoc comparisons in each analysis to reduce the risk of Type I errors. Finally, we wanted to investigate the associations between participants’ relationship satisfaction with their willingness to display affection in private and public contexts and their attitudes towards showing affection. Therefore, we planned to conduct correlation analyses using Pearson’s r coefficient.

## 3. Results

Before conducting analyses related to our study goals, we checked sex and age distributions in the collected samples. Chi-square analysis did not reveal sex differences between participants from different countries [χ^2^(2) = 3.11, *p* = .21]. Subsequently, in 2 (sex) × 3 (country) ANOVA with age as a dependent variable, we failed to detect both a main effect of sex [*F*(1, 455) = 1.01, *p* = .32; η^2^_p _< .01] and a main effect of country [*F*(2, 455) = 1.34, *p* = .26; η^2^_p _< .01]. On the other hand, we detected interaction [*F*(2, 455) = 3.32, *p* = .04; η^2^_p _= .01]. However, Bonferroni-Holm post hoc analysis did not reveal any differences between groups differing in terms of sex and country [|ts|(455) ≤ 2.42, *p*s ≥ .24, Cohen’s |*d*s| ≤ 0.37]. Therefore, our results showed *t*hat the groups of participants included in our study were comparable in terms of gender and age distribution. Table 1 contains descriptive statistics for all quantitative variables measured in our study.

**Table 1:** Means (standard deviations) for measured quantitative variables.

Variables	Indonesia sample			Nepal sample			Poland sample		
	Overall (*n *= 170)	Men (*n *= 62)	Women (*n *= 108)	Overall (*n *= 120)	Men (*n *= 56)	Women (*n *= 64)	Overall (*n *= 171)	Men (*n *= 72)	Women (*n *= 99)
**Age**	24.12 (5.88)	23.48 (6.23)	24.49 (5.66)	23.03 (4.29)	23.34 (5.31)	22.77 (3.16)	23.74 (4.84)	24.85 (5.54)	22.94 (4.12)
**Priv *PDA***	2.88 (0.69)	2.79 (0.77)	2.93 (0.64)	3.85 (1.04)	3.81 (1.04)	3.88 (1.05)	4.33 (0.66)	4.27 (0.74)	4.37 (0.61)
**Pub *PDA***	3.14 (0.78)	3.02 (0.85)	3.20 (0.73)	3.22 (1.02)	3.30 (1.00)	3.14 (1.05)	3.87 (0.77)	3.81 (0.87)	3.92 (0.70)
***Neg O***	2.72 (0.67)	2.73 (0.68)	2.72 (0.67)	2.25 (1.03)	2.31 (1.13)	2.20 (0.93)	1.85 (0.76)	1.90 (0.81)	1.82 (0.73)
***Neg B***	2.92 (0.81)	2.83 (0.86)	2.98 (0.78)	1.86 (0.96)	1.99 (1,00)	1.79 (0.92)	1.66 (0.67)	1.64 (0.65)	1.67 (0.69)
***RS***	3.08 (1.21)	3.13 (1.22)	3.06 (1.21)	3.82 (0.89)	3.79 (0.87)	3.84 (0.91)	3.43 (0.68)	3.42 (0.62)	3.43 (0.72)

Priv *PDA* = Private display of affection; Pub *PDA* = Public display of affection; *Neg O* = Negative opinions about people displaying affection in public; *Neg B* = Negative behaviors towards people displaying affection in public; *RS* = Relationship satisfaction.

**Fig 1:**
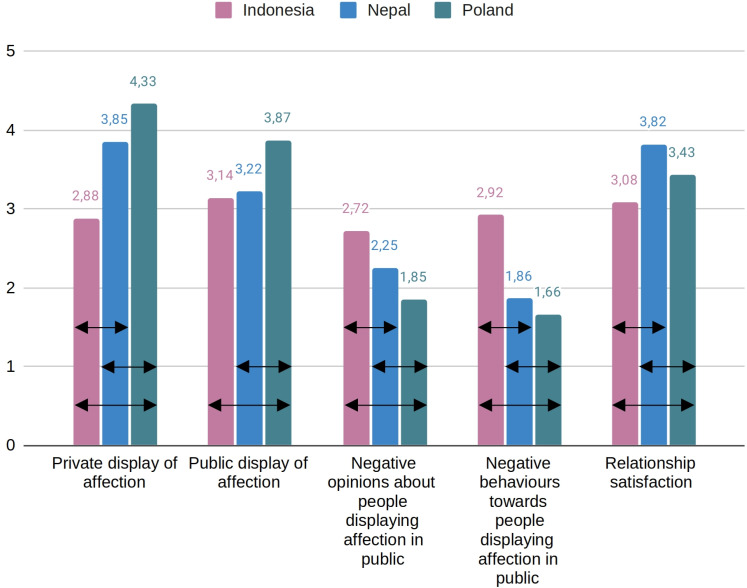
Main effects of country in ANOVA analyses. Arrows indicate effects significant at p < .05 in Bonferroni-Holm post-hoc tests.

We started with 2 (sex) × 3 (country) ANOVA with relationship satisfaction as a dependent variable. We revealed a main effect of country [*F*(2, 455) = 19.31, *p* < .01, η^2^_p _= .08; we show the main effects of country observed in all analyses in Figure 1]. Participants from Nepal were more satisfied with their relationships than participants from Indonesia and Poland [|ts|(288 and 289) ≥ 3.39, *p*s_Bonferroni-Holm_ < .01, |*d*s| ≥ 0.41] and par*t*icipants from Poland were more satisfied with their relationships than participants from Indonesia [t(339) = −3.13, *p*_Bonferroni-Holm_ < .01, *d* = −0.35]. We failed to de*t*ect other effects related to relationship satisfaction.

Next, we ran 2 (context of displaying affection) × 2 (sex) × 3 (country) mixed model ANOVA with willingness to display affection as a dependent variable. We revealed a main effect of the context of displaying affection [*F*(1, 455) = 76.96, *p* < .01, η^2^_p _< .15]; participants were more likely to show affection in a private context than in a public context [t(459) = 8.77, *p*_Bonferroni-Holm_ < .01, Cohen’s *d* = 0.34]. Moreover, we revealed a main effect of coun*t*ry [*F*(2, 455) = 88.21, *p* < .01, η^2^_p _< .28]; participants from Poland were more willing to show affection than participants from Indonesia and Nepal [ts(339 and 289) ≤ −6.21, *ps*_Bonferroni-Holm_ < .01, *d*s ≤ −0.68] and participants from Nepal were more willing to show affection *t*han participants from Indonesia [t(288) = −6.03, *p*_Bonferroni-Holm_ < .01, *d* = −0.67]. We also revealed interaction between the contex*t* of displaying affection and country [*F*(2, 455) = 72.77, *p* < .01, η^2^_p _< .24]. Bonferroni-Holm post hoc analyses revealed that almost all differences across contexts and countries were significant at *p* < .01 (|ds| ≥ 0.30); however, participants from Nepal were equally willing to display affection in a private context as participants from Poland in a public context [t(289) = −0.16, *p* = .87 *d* = −0.02] and participants from Indonesia and Nepal were equally willing to display affection in a public context [t(288) = −1.10, *p* = .55 *d* = −0.13]. We failed to detec*t* other effects related to the willingness to display affec*t*ion.

Subsequently, we ran 2 (type of attitude towards displaying affection in public) × 2 (sex) × 3 (country) mixed model ANOVA with negative attitude towards displaying affection in public as a dependent variable. We revealed a main effect of the type of attitude displaying affection in public [*F*(1, 455) = 16.65, *p* < .01, η^2^_p _< .04]; participants declared more negative opinions than negative behaviors towards people displaying affection in public [t(459) = 4.08, *p*_Bonferroni-Holm_ < .01, *d* = 0.16]. Moreover, we revealed a main effect of coun*t*ry [*F*(2, 455) = 86.19, *p* < .01, η^2^_p _< .28]; participants from Indonesia had more negative attitudes towards displaying affection in public than participants from Nepal and Poland [ts(288 and 339) ≥ 8.27, *ps*_Bonferroni-Holm_ < .01, *d*s ≥ 0.92] and participants from Nepal had more negative a*tt*itudes towards displaying affection in public than participants from Poland [t(289) = 3.58, *p*_Bonferroni-Holm_ < .01, *d* = 0.39]. We also revealed interaction between the type of a*t*titude towards displaying affection in public and country [F(2, 455) = 25.64, p < .01, η^2^_p_ < .10]. Bonferroni-Holm post hoc analyses revealed that almost all differences across contexts and countries were significant at *p* ≤ .03 (|ds| ≥ 0.29). However, participants from Poland declared negative opinions towards displaying affection in public comparable to negative behaviors towards people displaying affection in public among participants from Nepal [t(289) = −0.33, *p* = .75, *d* = −0.04]. We failed to detect other effects related to negative a*t*titudes towards displaying affection in public.

Finally, separately for each country, we correlated participants’ relationship satisfaction with their willingness to display affection in private and public contexts and their negative attitudes towards showing affection (see Table 2). In overall samples from each country, relationship satisfaction correlated with the willingness to display affection both in private and public contexts. On the other hand, correlations between relationship satisfaction and negative attitudes towards showing affection were differentiated: among Indonesian people, higher relationship satisfaction correlated with more negative opinions and more negative behaviors towards people displaying affection publicly. However, in the Polish sample, people more satisfied with their relationships displayed less negative behaviors towards people displaying affection in public. We failed to detect links between relationship satisfaction and negative attitudes towards showing affection among Nepali people.

**Table 2:** Correlations (Pearson’s rs) between relationship satisfaction and the Public and Private Romantic Display of Affection Scale.

	Relationship satisfaction								
Variables	Indonesia sample			Nepal sample			Poland sample		
	Overall	Men	Women	Overall	Men	Women	Overall	Men	Women
**Priv *PDA***	.31**	.38**	.27**	.19*	.24+	.15	.44**	.46**	.44**
**Pub *PDA***	.35**	.34**	.36**	.19*	.13	.25*	.20**	.20+	.21*
***Neg O***	.24**	.21+	.26**	−.10	.02	−.21*	−.10	−.01	−.16
**Neg *B***	.27**	.26*	.28**	.02	.20	−.13	−.19*	−.13	−.23*

Priv *PDA* = Private display of affection; Pub *PDA* = Public display of affection; *Neg O* = Negative opinions about people displaying affection in public; *Neg B* = Negative behaviors towards people displaying affection in public.

+ p < .10, * p < .05, ** p < .01.

## 4. Discussion

Displays of affection are common behaviors in romantic relationships [10]; however, they are regulated by cultural specificity [60,61] and religious norms [5]. People may also differ in their willingness to express affect depending on whether it occurs in a private or public context [7]. In our study, we compared tendencies for public and private displays of affection and attitudes towards people who display their affection publicly in Indonesian, Nepali, and Polish samples. In line with our hypothesis, we confirmed cross-cultural differences in romantic displays of affection. Participants from Poland were the most willing to show affection, followed by participants from Nepal, while those from Indonesia were the least willing. The observed differences may be influenced by cultural individualism, which plays a role in showing affection [36,39] and differs between Indonesia, Nepal, and Poland [40–42], along with the regulatory importance of the predominant religions in the countries compared. However, participants from Indonesia and Nepal demonstrated similar levels of willingness to display affection in public settings, despite differences in the dominant religions and the fact that the level of individualism in Nepal significantly exceeds the level of individualism in Indonesia [40,41]. On the other hand, there may also be other, more specific cultural norms or regional traditions that influence the willingness to display affection among Indonesian and Nepali people.

The *PPRDAS* questionnaire [7] includes expressions of affection such as holding hands, hugging, kissing, and verbal declarations of love. Previous studies confirmed cultural differences in both verbal and nonverbal expressions of affection. For instance, studies comparing American and Chinese students found differences in the methods used to express appreciation in romantic relationships. Americans relied equally on verbal and nonverbal methods, while Chinese participants significantly preferred nonverbal methods. Additionally, the Chinese used more indirect methods than Americans [60]. Moreover, studies have shown that verbal expressions of love are culturally dependent [61]. For example, Americans and East Asians (China, Japan, and South Korea) differ in the expressions associated with love in marriage. Americans were more likely than East Asians to perform acts of consideration as expressions of love (e.g., focusing on others, showing interest, doing things out of the ordinary, and making oneself attractive) toward their spouse. Interestingly, not all studies show cultural differences. For instance, Kline et al. [62] did not show cross-cultural differences in terms of oral/written direct expressions of love or physical contact.

Participants from Indonesia had the most negative attitudes towards displaying affection in public, followed by those from Nepal, while participants from Poland showed the least negative attitudes. Attitudes towards public displays of romantic affection influence one’s willingness to express affection [7], translating into less affectionate romantic behavior in Indonesia and Nepal. In addition to factors such as individualism and religion, conservatism may play a role here. Affective touch is relatively higher in warmer, less conservative, and less religious countries and among younger people, women, and liberal people [5]. Also, studies conducted in another highly conservative country, the Philippines, showed that the majority of participants agreed with the statement “Students engage in PDA (public displays of affection) because they lack discipline and moral values.” [63 p. 80].

As we hypothesized, both private and public displays of affection were positively correlated with relationship satisfaction across samples from all three countries, although these associations were weakest in Nepal. Participants from Nepal reported the highest level of satisfaction with their relationships, followed by participants from Poland, while those from Indonesia were the least satisfied. This result is particularly interesting considering that romantic displays of affection (both private and public) were more frequent in Poland, and the Polish participants had the most positive attitudes towards public displays of affection. Additionally, in Nepal, private displays of affection were associated with relationship satisfaction among men, whereas among women, it was public displays of affection that positively correlated with relationship satisfaction. In the other two countries, the correlations were similar for men and women. However, relationship satisfaction is not solely determined by the level of affection within the relationship [64]. Relationship satisfaction has multiple sources and correlates, such as ethnicity, culture, country, socioeconomic conditions, age, relationship duration, and having children [64]. One of the causes of lower relationship satisfaction scores among participants from Indonesia may be their reduced level of affection, even if this is expressed in a private context. Interestingly, in Indonesia, scores for public display of affection scores were slightly higher than for private ones. This may indicate a generally lower level of passion among participants from that country. On the other hand, some cross-cultural studies suggest that passion may be perceived differently across cultures. For example, although aspects such as intimacy and commitment are considered important in both China and the United States, passion is perceived as much more important by American couples than by Chinese couples [65].

We also observed a negative correlation between attitudes towards public displays of affection and relationship satisfaction in Poland and Nepal. Similar results have been reported in previous studies in Poland, where people not engaged in romantic relationships exhibited the highest levels of negative attitudes towards public displays of affection [7]. These results suggest that an unsatisfied need for affection may generate more negative reactions towards people who express their affection publicly. However, our results collected in Indonesia showed that participants more satisfied with their romantic relationships had more negative attitudes towards other people engaging in public displays of affection. This result warrants further investigation and the exploration of potential mediators for this relationship.

### 4.1. Limitations and directions for future research

Our study has some limitations. Firstly, we focused exclusively on heterosexual relationships due to cultural constraints surrounding public displays of affection in relationships that are less socially accepted. Numerous studies highlight the limitations and concerns associated with publicly expressing romantic affection in less socially accepted relationships, such as same-sex relationships [66] and interracial relationships [14]. In future studies, it would be valuable to collect data from LGBTQ+ people, as the level of acceptance for non-heterosexual relationships varies significantly across different countries. Secondly, future research should consider exploring potential mediators of the relationship between affection and greater relationship satisfaction; for instance, conflict resolution styles or stress-coping strategies could serve as culturally dependent mediators [see 67]. Thirdly, future studies may use more precise measures of expressions of affection, such as holding hands, hugging, kissing, and verbal declarations of love because previous studies showed cultural differences in preferred expressions of affection, both verbal and nonverbal. For instance, Americans relied equally on verbal and nonverbal methods, while Chinese participants significantly preferred nonverbal methods [60]. Moreover, studies have shown that verbal expressions of love are culturally dependent [61]. For example, Americans and East Asians (e.g., people from China, Japan, and South Korea) differ in the expressions associated with love in marriage. Americans were more likely than East Asians to perform acts of consideration as expressions of love (e.g., focusing on others, showing interest, doing things out of the ordinary, and making oneself attractive) towards their spouse. Fourthly, we used a single-item scale to measure relationship satisfaction, the reliability of which could not be determined. However, studies have shown that such scales are valid and allow researchers to collect data with little effort from participants [68,69]. Finally, future research should include cultures with a high level of individualism, such as the United States, particularly to allow for comparisons of the associations between displays of affection and relationship satisfaction.

## 5. Conclusions

Our study highlights the significant role of cultural norms in shaping public and private displays of romantic affection and connections between public and private displays of romantic affection with relationship satisfaction. Our findings reveal cultural differences between Indonesia, Nepal, and Poland. Importantly, a consistent positive association was observed between displays of affection and relationship satisfaction across all three countries, reinforcing the universal importance of affectionate behaviors in romantic relationships. These findings emphasize the need to consider cultural factors when examining relationship dynamics and suggest that promoting affectionate behaviors tailored to cultural contexts may enhance relationship satisfaction.

Beyond its empirical contributions, our study offers theoretical and practical insights. The consistent positive link between affectionate behaviors and relationship satisfaction across three cultures supports key assumptions of Affection Exchange Theory. In practice, these findings may inform relationship counseling, suggesting that encouraging appropriate expressions of affection, adjusted to cultural sensitivities, can strengthen romantic bonds. Awareness of cultural norms around public affection can also help counselors, HR professionals, and policymakers promote well-being and respectful interpersonal dynamics. Moreover, sectors such as tourism and hospitality could use these insights to educate visitors about local norms regarding display of affection in public places and encourage culturally sensitive behavior, thus promoting mutual respect and positive intercultural experiences.

## Supporting information

S1 Appendix Inclusivity in global research questionnaire. (DOCX)
