# Supplementary material for: To hug or not to hug? Public and private displays of affection and relationship satisfaction among people from Indonesia, Nepal, and Poland
Source: PLoS One. 2025 Jun 25;20(6):e0326115. doi: 10.1371/journal.pone.0326115 (PMC12193689; doi:10.1371/journal.pone.0326115)
Supplement: S1 Appendix — (DOCX) [file pone.0326115.s001.docx]

**Research questionnaire**

The study is carried out by the Interdisciplinary Team of the Faculty of Social Sciences at the University of Silesia. It should not take you more than 10 minutes to complete the questionnaire.

Thank you.

Before proceeding to complete the questionnaires, please read the following information about the nature of the survey.

**1. Survey aim:**

The aim of the survey is to learn about private and public displays of affection in romantic relationships.

**2. Respondent’s role:**

As part of the survey, we ask the respondents to provide demographic data, as well as ask questions about attitudes towards displays of affection in private and public situations.

**3. Data use:**

The data obtained in the survey will serve scientific purposes and will only be used within the framework of the study. The individual responses will not be made available to anyone, and the research conclusions will be drawn on the basis of the aggregate survey results.

**4. Voluntary and confidential nature of the study:**

The survey is fully anonymous, meaning that no data will be collected that could be used to identify the respondents. The data collected is confidential, with access limited to the person conducting the study and the person supervising the study. Respondents are allowed to withdraw from the study at any time and opt out of submitting their answers to the survey questions.

**5. Risks related to the study:**

The survey may include questions concerning the intimate sphere of sexual life. Your participation in the study is of great value, but above all remember you should have psychological comfort and that you can opt out of the study at any time.

**6. Survey duration and conditions:**

The estimated survey time is 20–25 minutes.

Having read the above information, do you give your informed consent to participate in my survey?

• Yes, I agree to participate in the survey and I confirm that I am of legal age.

• No, I do not agree to participate in the survey or I am not of legal age.

| 1. Age........... | 1. Gender:    1. Woman    2. Man    3. Non-binary    4. I don’t want to define myself |
| --- | --- |
| 1. Education:    1. elementary,    2. vocational,    3. secondary,    4. higher. | 1. Place of residence:   a) countryside,  b) town/city with a population of 10 to 100 thousand  c) city with a population of 100 to 500 thousand,  d) city with a population of over 500 thousand. |
| 1. Relationships:   a) I’m single,  b) I’ve been in an informal relationship for less than one year,  c) I’ve been in an informal relationship for more than one year,  d) I’m married,  e) other,  please specify .......................................... | |

1. Rate your relationship on the following scale:

| **0** | **1** | **2** | **3** | **4** | **5** |
| --- | --- | --- | --- | --- | --- |
| Not applicable | Very unsuccessful | Unsuccessful | I have no opinion | Successful | Very successful |

**Public and Private Romantic Display of Affection Scale**

Presented below is a number of statements related to public and private displays of affection. Indicate how well each statement matches your behaviour. There are no good or bad answers. Try to make your answers honest and sincere. Put your answers in the left column. Use the scale given below:

| **1** | **2** | **3** | **4** | **5** |
| --- | --- | --- | --- | --- |
| Completely does not match my behaviour | Does not match my behaviour | Matches my behaviour a little | Matches my behaviour | Completely matches my behaviour |

|  | 1. I like holding my partner’s hand while at home (e.g. watching a film). |
| --- | --- |
|  | 2. I like walking in public places holding my partner’s hand. |
|  | 3. Couples holding hands in public places annoy me. |
|  | 4. I sometimes admonish couples whose behaviour is inappropriate for public places (display of passion). |
|  | 5. I like to hug my partner when we are home alone (e.g. sitting on the sofa). |
|  | 6. If I walked with my partner in the park, I would hold his/her hand. |
|  | 7. Couples kissing in public places annoy me. |
|  | 8. I sometimes admonish couples who kiss passionately in public places. |
|  | 9. I like to fleetingly (briefly) kiss my partner when we are home alone. |
|  | 10. If I spent time in the park and sat on a bench, I would hug my partner. |
|  | 11. Couples hugging in public places annoy me. |
|  | 12. I sometimes make loud comments on couples kissing passionately in public places. |
|  | 13. I often kiss my partner passionately during the day when we are home alone. |
|  | 14. I sometimes fleetingly (briefly) kiss my partner in public places. |
|  | 15. Couples kissing passionately in public places annoy me. |
|  | 16. I sometimes make loud comments on couples hugging passionately in public places. |
|  | 17. I often tell my partner that I love him/her when we are home alone. |
|  | 18. I often tell my partner in the presence of other people (family, friends) that I love him/her. |
|  | 19. I sometimes ridicule couples kissing passionately in public places. |
